# A Case Presentation of a Rare Pelvic Interdigitating Dendritic Cell Sarcoma

**DOI:** 10.7759/cureus.54220

**Published:** 2024-02-14

**Authors:** Tasciana T Gordon, Trent Cross, John Liu

**Affiliations:** 1 General Surgery, Mater Hospital Brisbane, Brisbane, AUS; 2 Pathology, Mater Pathology, Mater Hospital Brisbane, Brisbane, AUS

**Keywords:** interdigitating dendritic cell sarcoma, dendritic cell, idcs, pelvic sarcoma, sarcoma

## Abstract

Sarcoma is a rare type of cancer that arises from connective tissue. Interdigitating dendritic cell sarcoma (IDCS) is a rare neoplasm of dendritic cell origin. IDCS arises from interdigitating dendritic cells found in the T-cell regions of secondary lymphoid tissues. Due to the rare nature of IDCS diagnosis, management can be difficult. Often, the diagnosis is delayed due to a lack of symptoms and signs. Here, we describe a case of a 34-year-old female patient who presented with an incidental finding of a left sidewall pelvic mass later to be confirmed on biopsy as an IDCS.

## Introduction

Interdigitating dendritic cell sarcoma (IDCS) is an extremely rare neoplasm with approximately 100 cases reported in published literature [1]. IDCS is a sarcoma that arises in dendritic cells that mostly occur in lymph nodes and rarely in extranodal sites [1]. Dendritic cells participate in the immune system as antigen-presenting cells, with four different types of dendritic cells existing in lymph nodes. These are histiocytic, fibroblastic, interdigitating, and follicular dendritic cells [1]. Their main function is the presentation of antigens and the generation and regulation of the germinal center [1,2]. This is done by presenting antigens for B-cells and stimulating B-cell proliferation and differentiation. Interdigitating dendritic cells are found in the T-cell areas of secondary lymphoid tissues [1-4].

Dendritic cell neoplasms are rare tumors and were previously classified as lymphomas, sarcomas, or histiocytic neoplasms. The World Health Organization (WHO) has since developed a classification for dendritic cell neoplasms into five groups: Langerhans cell histiocytosis (LCH), Langerhans cell sarcoma (LCS), interdigitating dendritic cell sarcoma (IDCS), follicular dendritic cell sarcoma (FDCS), and dendritic cell sarcoma not otherwise specified (DCSNOS) [5]. This differentiation has allowed for improved analysis of cases with a review of management and treatment.

Here, we report a case of nodal IDCS presenting in the pelvis.

## Case presentation

A 34-year-old female presented with upper abdominal pain and was initially investigated for biliary colic. She underwent a radiographic investigation with an ultrasound and then computed tomography (CT) of the abdomen that showed a large left sidewall pelvic mass. She had no medical history, no significant family history, and was a non-smoker. 

**Figure 1:**
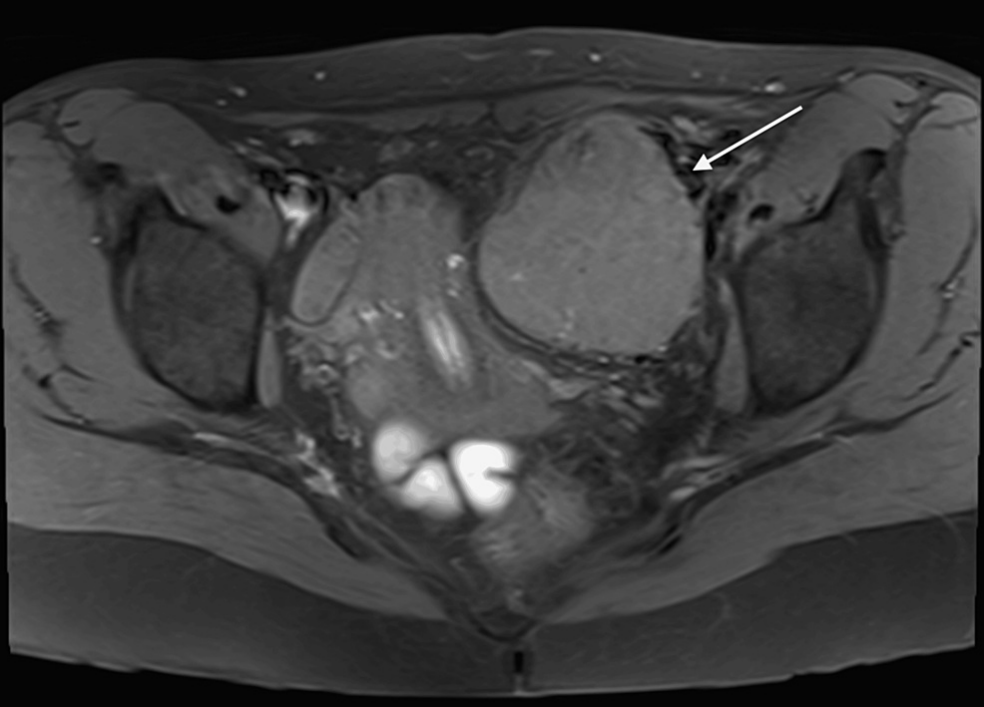
MRI T1 axial pelvis showing the left pelvic sidewall (IDCS) (white arrow) MRI: Magnetic resonance imaging; IDCS: Interdigitating dendritic cell sarcoma

**Figure 2:**
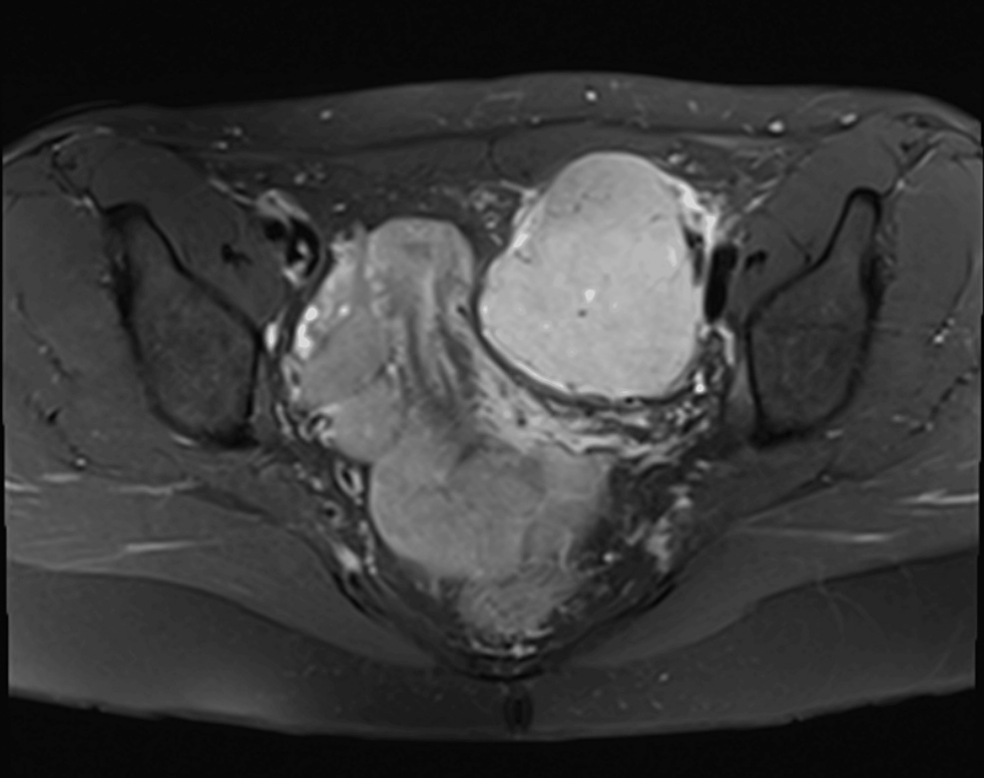
MRI pelvis showing the encapsulated left pelvic sidewall (IDCS) MRI: Magnetic resonance imaging; IDCS: Interdigitating dendritic cell sarcoma

A pelvic magnetic resonance imaging (MRI) was conducted to further classify the pelvic mass. The mass was noted to be separate from the left ovary and appeared to be outside the peritoneal cavity within the left pelvic sidewall immediately medial to the external iliac vessels. Measuring 66x60x76 mm, there was no invasion into the anterior abdominal wall muscles or other pelvic structures (Figures 1-2). 

**Figure 3:**
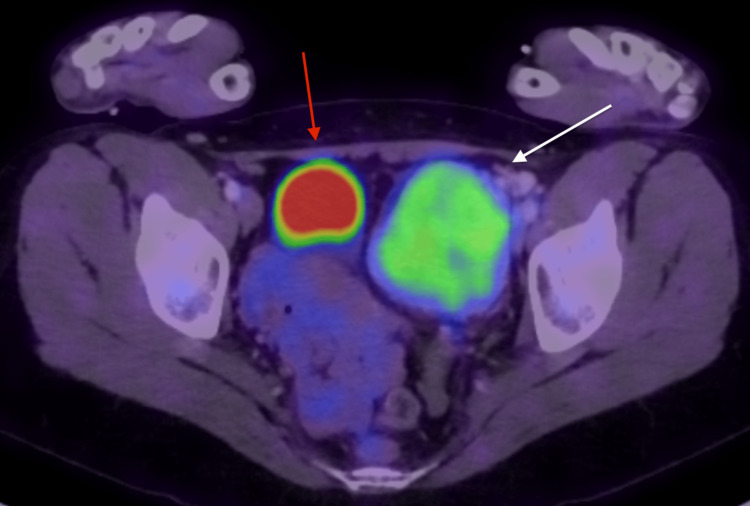
PET/CT showing FDG-avid left pelvic wall mass (white arrow) and bladder (red arrow) PET/CT: Positron emission tomography/computed tomography; FDG: Fludeoxyglucose

Positron emission tomography/computed tomography (PET/CT) was performed and showed a large mass in the left iliac fossa that was homogenous with moderate fludeoxyglucose (FDG) avidity (Figure 3). It was a solitary lesion with no other synchronous or distal lesions elsewhere.

Multiple biopsies were sampled via CT guidance to assist with the characterization of the lesion and surgical planning. The biopsies were reviewed by multiple pathologists and referred to a hematopathologist for a third opinion. The biopsy showed a sclerosing lesion associated with lymphoplasmacytic infiltrate. The case was discussed at a sarcoma multidisciplinary team meeting, and the patient underwent a laparotomy, wide local excision, and selected lymph node dissection. 

**Figure 4:**
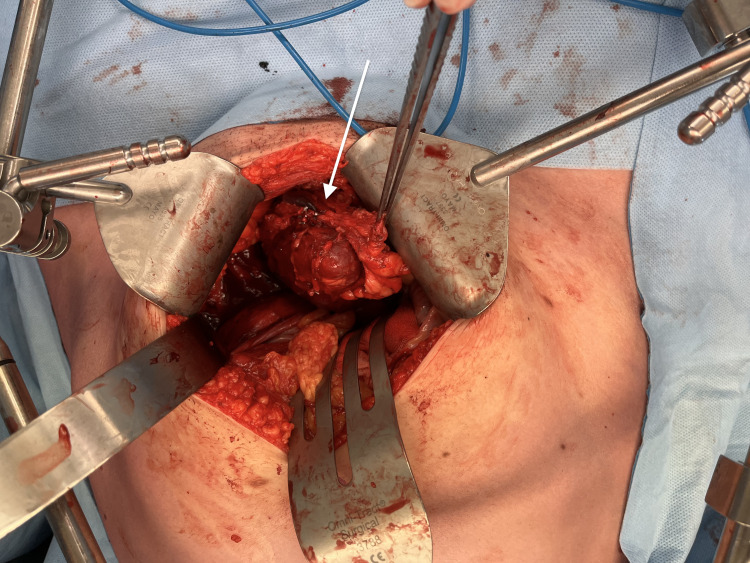
Intraoperative clinical photograph of left pelvic mass (IDCS) (white arrow) IDCS: Interdigitating dendritic cell sarcoma

**Figure 5:**
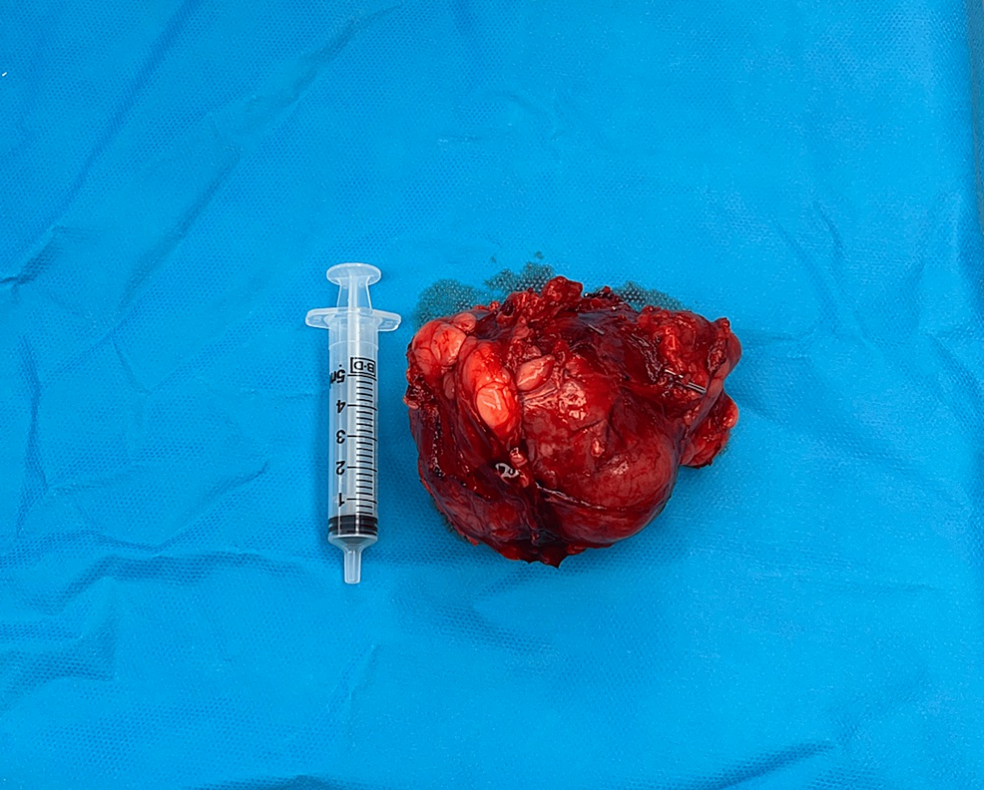
Photograph of left pelvic mass (IDCS) specimen following wide local excision IDCS: Interdigitating dendritic cell sarcoma

The macroscopic specimen measured 114x73x58 mm, weighing 190 g (Figures 4-5). 

**Figure 6:**
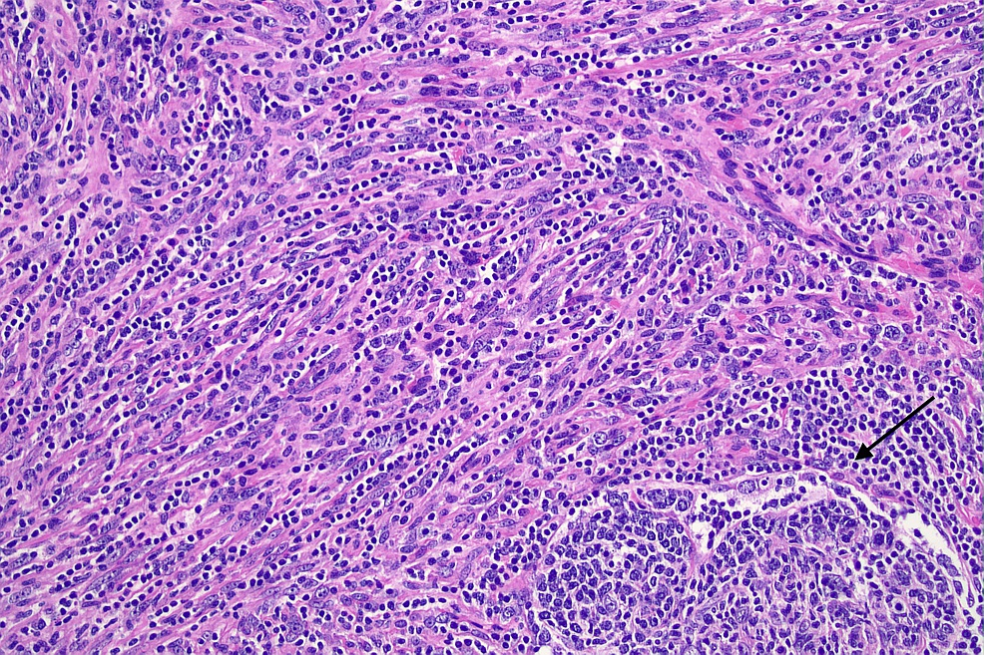
Histological slide showing loose fascicles of pleomorphic spindle cells with associated lymphocytes. A residual lymphoid follicle is present at the bottom right (black arrow) (H&E, 200X) H&E: Hematoxylin & eosin

Histology showed a highly unusual neoplasm comprising loose fascicles and whorls of spindle cells with an infiltrate between lymphoid follicles, associated with a prominent lymphoplasmacytic infiltrate. The neoplasm had arisen in a lymph node with normal nodal tissue around the periphery. On immunohistochemistry, the spindle cells were focally positive for S100, SMA, CD31, CD138 and negative for CD21, CD23, CD3, CD20, SOX10, BRAF, ALK. This is consistent with an IDCS, 93 mm in size (Figure 6).

## Discussion

IDCS is a rare neoplasm with approximately 100 cases reported in the English literature [1,4]. There have been nodal cases described, however, no pelvic nodal cases like this case have been reported [1-4]. This case is a rare presentation of a pelvic nodal IDCS. 

IDCS was first reported in 1981 by Feltkamp and coworkers where a patient was found to have a mass in the mediastinum. There is a slight male predominance (1.38:1), and the median age of diagnosis is 56.5 years [1-4]. The clinical presentation will vary depending on the site of pathology. The most common pathology is an isolated lymph node [1-4]. Other sites of involvement include extranodal lesions including the liver, gastrointestinal tract, lung, spleen, skin, nasopharynx, kidney, bladder, ovary, and testis [1]. Most cases present following an incidental finding of an abnormally enlarged lymph node. Extranodal cases present more aggressively due to the lack of symptoms and aggressive progression of the disease. This may present as haemoperitoneum or bowel obstruction in cases with gastrointestinal IDCS [1,5-7].

The etiology of IDCS remains unclear. There are a few postulated theories with hematopoietic stem cells, migration of Langerhans cells, and infectious origins such as Epstein-Barr virus or human herpesvirus [3-5]. However, viral etiology is more likely to be associated with follicular dendritic cell sarcomas rather than IDCS. There may also be an association with immunosuppression with tacrolimus or pimecrolimus [3-6]. These calcineurin inhibitors are known to dampen the T-cell response with dysregulation of the immune system, which can facilitate the malignant transformation of interdigitating dendritic cells. There is a similarity in pathogenesis with that of low-grade B-cell lymphoma due to trans-differentiation where B-cells change phenotypically but remain in genotypical features [3-7]. There is a poor prognosis associated with hematopoietic stem cells, which appear to be the most common in the gastrointestinal IDCS. This leads to patients presenting with hemoperitoneum or bowel obstruction [7].

Dendritic cell sarcomas should be differentiated from other dendritic neoplasms. The most common morphological pattern is fascicular arrangement [2,3]. The cells are most often spindle in nature and are less often epithelioid or markedly pleomorphic. The diagnosis cannot be made with clinical and light microscopy alone. Immunohistochemistry helps in differentiating the lesion with tumor cells typically positive for S100, myeloid cell marker CD68, and leukocyte common antigen [2,3]. There have also been some commonalities described with negative staining for CD1a, CD3, CD20, HMB45, or cytokeratin [2,3].

Our case underwent a laparotomy, wide local excision, and selective lymph node dissection following discussion at a multidisciplinary sarcoma meeting. There were clear margins and the disease was isolated to a single lymph node measuring 93 mm. The patient was discharged on the fourth day postoperatively with no complications. Given the clearance and no evidence of hematogenous spread on PET/CT, no adjuvant therapy was administered. She was monitored with a follow-up MRI pelvis at three months postoperatively and continues to be monitored with surveillance six-monthly chest X-rays. 

Consistent standard guidelines for treatment have not been established [1-4,7]. Surgery is the mainstay of treatment with or without adjuvant chemotherapy and radiotherapy [1,5-7]. A larger case series would need to be developed to establish any statistical significance in treatment regimes. Utilizing multidisciplinary team meetings ensures these patients have an improved outcome [7]. The risk of local recurrence is related to the size, location, and margins of surgical excision [5-7]. Although there is no standardized follow-up protocol, recommendations include surveillance for local recurrence and pulmonary metastases. This can be done with CT imaging or chest X-ray. 

## Conclusions

IDCS is a rare, aggressive malignancy that is often misdiagnosed. Having an awareness of the morphology and a high degree of suspicion if there is a single FDG-avid node may lead to earlier diagnosis and treatment. The diagnosis needs to include immunohistochemistry, clinical, and light microscopy. There is a limited number of cases presented in the literature and consensus on adjuvant therapy is lacking. However, surgery remains the mainstay of treatment. Follow-up should include surveillance of the local region and pulmonary metastases as this is the most common site for metastatic disease. Publishing these cases as they present will help determine patterns and create awareness amongst surgeons and pathologists. This will also assist with earlier diagnosis and management of patients presenting with IDCS. Reviewing these patients over a longer period will also help determine a protocol for follow-up specific to IDCS.
